# Impact of Interleukin-17 Inhibitor Therapy on Arterial Intima-media Thickness among Severe Psoriatic Patients

**DOI:** 10.3390/life11090919

**Published:** 2021-09-05

**Authors:** Éva Anna Piros, Ákos Szabó, Fanni Rencz, Valentin Brodszky, Klára Szalai, Noémi Galajda, Bálint Szilveszter, Edit Dósa, Béla Merkely, Péter Holló

**Affiliations:** 1 Department of Dermatology, Venereology and Dermatooncology, Semmelweis University, 1085 Budapest, Hungary; szalai.klara@med.semmelweis-univ.hu (K.S.); galajda.noemi@med.semmelweis-univ.hu (N.G.); hollo.peter@med.semmelweis-univ.hu (P.H.); 2 Rácz Károly Doctoral School of Clinical Medicine, Semmelweis University, 1085 Budapest, Hungary; akos.szabo@uni-corvinus.hu; 3 Department of Health Economics, Corvinus University, 1093 Budapest, Hungary; fanni.rencz@uni-corvinus.hu (F.R.); valentin.brodszky@uni-corvinus.hu (V.B.); 4 Heart and Vascular Center, MTA-SE Cardiovascular Imaging Research Group, Semmelweis University, 1122 Budapest, Hungary; szilveszter.balint@med.semmelweis-univ.hu (B.S.); merkely.bela@cardio.sote.hu (B.M.); 5 Heart and Vascular Center, Department of Interventional Radiology, Semmelweis University, 1122 Budapest, Hungary; dosa.edit@med.semmelweis-univ.hu; 6 Hungarian Vascular Radiology Research Group, 1082 Budapest, Hungary

**Keywords:** psoriasis, interleukin-17 inhibitor, intima-media thickness, inflammation, atherosclerosis

## Abstract

Background: Psoriasis is frequently accompanied by cardiovascular diseases based on the shared immunopathogenic pathway. Authors determined the effect of interleukin (IL)-17 inhibitor therapy on arterial intima-media thickness (IMT) among severe psoriatic patients. Methods: Thirty-one severe psoriatic patients were enrolled. Twenty received secukinumab and 11 received ixekizumab. Before treatment initiation and after 6 months, the carotid-brachial-femoral IMT, the Psoriasis Area Severity Index (PASI), the Dermatology Life Quality of Index (DLQI) and the EuroQol Visual Analogue Scale (EQ VAS) were evaluated. Results: After 6 months, significant ameliorations were observed in PASI (*p* < 0.001) from 18 to 0, in DLQI (*p* < 0.001) from 17 to 0, in EQ VAS (*p* < 0.001) from 60 to 90, in right carotid IMT (*p* < 0.001) from 1.1 mm to 0.8 mm, in left carotid IMT (*p* < 0.001) from 1.1 mm to 0.7 mm, in right brachial IMT (p < 0.001) from 0.75 mm to 0.6 mm, in left brachial IMT (*p* < 0.001) from 0.8 mm to 0.5 mm, in right femoral IMT (*p* < 0.001) from 0.9 mm to 0.7 mm and in left femoral IMT (*p* < 0.001) from 0.8 mm to 0.7 mm. Conclusions: By reducing the inflammation of the vascular wall, anti-IL-17 therapy may have a beneficial long-term effect on cardiovascular complications of systemic inflammation.

## 1. Introduction

Psoriasis is a chronic immune-mediated autoinflammatory disorder that has a genetic predisposition (PSORS 1–9 genes) and develops along immunopathogenic pathways. Regarding population-based data, the worldwide prevalence of the disease is approximately 2–3% [1].

Psoriasis mainly affects the skin and joints (psoriatic arthritis), but due to the systemic inflammation, referred to as ‘psoriatic march’, it can greatly impact the cardiovascular system [2]. The shared self-reinforcing immunopathogenic pathway is responsible for the strong association between psoriasis and cardiovascular diseases (CVDs). The central cytokines belong to the T-helper type 1 (Th1) line and to the T-helper type 17 (Th17)/ interleukin-23 (IL-23) axis, like the tumour necrosis factor (TNF), interferon-gamma (IFN-γ), IL-12, IL-17A-F, IL-22, and IL-23 [3]. Systemic inflammation, which causes persisting symptoms of psoriasis, also has an influence on vasculature, and through inflammation of the arterial wall, it can lead to atherosclerotic plaque formation and progression. Atherosclerosis can then manifest in major cardiac events (MACEs), such as myocardial infarction (MI) and stroke [4,5].

Severe psoriatic patients have an increased risk of stroke (HR, 1.38; 95% CI, 1.20–1.60), MI (HR, 1.70; 95% CI, 1.18–2.43), and cardiovascular death (HR, 1.37; 95% CI, 1.13–1.67) [6]. The life expectancy of untreated severe psoriatic patients is approximately 5 years shorter than that of non-psoriatic healthy individuals [7].

Intima-media thickness (IMT) is an indicator of early atherosclerosis and can easily be measured by ultrasound examination of the large arteries: common carotid, brachial, and common femoral [8]. Several studies have demonstrated increased carotid IMT (cIMT) among psoriatic patients compared to non-psoriatic healthy controls [9,10]. Femoral IMT (fIMT) is a more informative predictor of subclinical atherosclerosis than cIMT [11,12]. Existing research has found conflicting results about the effect of systemic antipsoriatic therapies on cIMT. There are studies where moderation of psoriatic inflammation with systemic antipsoriatic treatments can ameliorate IMT values. In our previous study, we observed a significant improvement of the carotid and brachial IMT (bIMT) values among moderate-to-severe psoriatic patients after 6 months of TNF inhibitor treatment [9,13]. In contrast, other studies found no significant improvement in cIMT values by using ustekinumab, secukinumab, ixekizumab, and adalimumab therapies [14,15].

The role of IL-17 and its link to the vascular endothelial cell dysfunction, and subsequently the progression of atherosclerosis, is still under research. The literature is controversial and some studies show that IL-17 is proatherogenic and its inhibition may reduce atherosclerosis, while other studies show that IL-17 has an atheroprotective effect. This discrepancy is probably due to differences in mouse models and experimental design. The activation of the IL-17A receptor, which is omnipresently expressed in the vessel wall, induces the production of TNF, IL-1β, intercellular adhesion molecule 1 (ICAM-1), thus providing an association with the pathogenesis of atheroslcerosis [16]. In ApoE(−/−) mice with atherosclerosis, the expression of IL-17 and retinoic acid-related orphan receptor γt (ROR γt) was substantially higher in the arterial wall with plaque than in the arterial wall without plaque. Treatment of ApoE(−/−) mice with a neutralizing anti-IL-17 antibody inhibited the development of atherosclerotic plaque, whereas the application of recombinant IL-17 significantly promoted the formation of the atherosclerotic plaque [17]. IL-17 also participates in the development of myocardial fibrosis through the PKCß/ERK1/2/NF-κB signaling pathways and promotes endothelial dysfunction and angiotensin II-induced hypertension [18,19]. On the other hand, results from a prospective, multicentre French registry with 981 patients with acute MI show that low IL-17 and high soluble vascular cell adhesion molecule 1 (VCAM-1) serum levels are associated with a higher risk of cardiac death and recurrent MI [20].

One of the latest systemic biologic antipsoriatic therapies is the IL-17 antagonist group, which targets the Th17/IL-23 axis. Four drugs are currently registered in this group for psoriasis treatment: ixekizumab, secukinumab, brodalumab, and bimekizumab. Although data are limited, there are some studies in the literature about these drugs’ effect on coronary atherosclerosis. After one year of treatment, Elnabawi et al. found the greatest reduction in coronary plaque burden in the anti-IL-17-treated group compared to the anti-TNF, anti-IL-12/23, and topical/phototherapy-treated groups [21]. In a Japanese case study, coronary constriction and interruption significantly improved after 2 years of secukinumab treatment [22]. Further investigations are needed to examine these drugs’ effects on the early stage of atherosclerosis.

In this self-controlled prospective study, our aim was to determine the long-term (6-month) effect of anti-IL-17 therapies (secukinumab and ixekizumab) on arterial (carotid, brachial, and femoral) IMT, skin symptoms, and quality of life among adult patients with severe plaque-type psoriasis.

## 2. Materials and Methods

This study was approved by the Regional Institutional Scientific and Research Committee of Semmelweis University, Budapest, Hungary (approval number: 154/2019; date of approval: 12 September 2019) and meets all the requirements of the Declaration of Helsinki. All enrolled patients were informed about the specific study procedures and signed the patient consent forms. The study was conducted between September 2019 and February 2021 in the Department of Dermatology, Venereology and Dermatooncology of Semmelweis University, Budapest, Hungary. All patients were followed-up for 6 months. Statistical analyses were assessed at the Department of Health Economics, Corvinus University of Budapest, Hungary.

### 2.1. Assessment of Clinical and Ultrasonographic Responsiveness

Thirty-one patients (13 women, 18 men; median age, 49 years (interquartile range, 37–62 years)) with severe plaque-type psoriasis were enrolled in the study.

Disease severity and quality of life (QoL) outcomes were determined by three severity rating scales that are frequently used in dermatology: Psoriasis Area Severity Index (PASI), Dermatology Life Quality Index (DLQI), and EuroQol Visual Analogue Scale (EQ VAS) [23,24]. Before initiation of anti-IL-17 therapy, the severity of skin symptoms and the patients’ health-related quality of life were assessed with the use of these scales. Severe psoriatic patients (PASI > 10 and DLQI > 10) were enrolled. Moreover, patients were included who received ineffective topical and/or systemic treatment prior to anti-IL-17 treatment [25]. ‘High disease activity’ refers to ongoing systemic inflammation. Before the initiation of the applied therapy, there was an average 3 month “wash-out” period to minimalize the effect of the previously used treatment on the baseline IMT values. IMT was measured manually on longitudinal B-mode ultrasound images obtained with a linear array transducer (3–13 MHz; Hitachi Preirus Al Vision, Hitachi Medical Corp., Tokyo, Japan) by an experienced radiologist (KS). The sites of IMT measurement were the common carotid artery, the mid-third of the brachial artery, and the common femoral artery on both sides. Measurements were performed on the dorsal wall of the arteries, and for each artery, the highest IMT value was recorded. At the beginning of the study, no fIMT measurement was performed.

Plaque—localized anywhere in the aforementioned arteries—was defined as a focal structure that protruded at least 0.5 mm into the vessel lumen, and/or measured 50% more than the adjacent IMT, and/or had an IMT > 1.5 mm [26]. Plaques were divided into non-calcified and calcified types. In the case of calcified plaques, acoustic shadowing was present. The total plaque area was also determined. All images were traced in the plane in which the plaque appeared the largest, and the ultrasound machine’s own software program calculated the cross-sectional area. Adding the area of each plaque gave the total plaque area [27].

Six months after the initiation of therapies, clinical (PASI, DLQI, EQ VAS) and ultrasound examinations were repeated. The 6-month scans were carried out by the same radiologist, and pre- and posttherapy measurements were taken in the same segment of the arteries.

### 2.2. Administration of Anti-IL-17 Therapies

Drugs were administered at the labelled dose for psoriasis: secukinumab 300 mg in weeks 0, 1, 2, 3 and 4 and then every 4 weeks, and ixekizumab 160 mg in week 0, then 80 mg in weeks 2, 4, 6, 8, 10 and 12, and then 80 mg every 4 weeks.

### 2.3. Evaluation of Data

Taking into consideration the small sample size and skewed distribution of data, the median values and interquartile ranges (IQRs) of the examined parameters (PASI, DLQI, EQ VAS scales and carotid-brachial-femoral IMT values) were calculated before and 6 months after the initiation of the therapies. Differences between the initial and the 6-month follow-up periods were analysed with the nonparametric Wilcoxon signed-rank test. All analyses were carried out using IBM SPSS Statistics version 25.0 software (IBM Corp, Armonk, NY, USA). *p* < 0.05 was considered statistically significant.

## 3. Results

### 3.1. Patient Characteristics

Baseline patients’ characteristics can be seen in Table 1.

The median disease duration time at the time of enrolment was 24 (IQR, 16–28) years. Four of the 31 patients (12.9%) were bionaive, which means that there was no previous systemic biologic therapy in the patients’ history. Among their previous treatments were topical corticosteroid therapies and methotrexate or acitretin. In the rest of the cases (87.1%), anti-IL-17 treatments were administered as second- or third-line systemic biologic antipsoriatic treatments. The enrolled patients had pronounced skin and/or joint symptoms for at least 3 months, supporting the existence of systemic inflammation, which was expressed during therapy changes. In our study population, patients had very severe skin symptoms: 12 (38.7%) had PASI ≥ 20 and nine (29%) had DLQI ≥ 25. Among the previously used systemic therapies were methotrexate (MTX), cyclosporine, acitretin, TNF blockers (etanercept, infliximab, adalimumab), and anti-IL-12/23 antibody (ustekinumab). For these patients, switching was necessary due to the loss of the drug effect or primary inefficacy. Eleven patients (35.5%) received ixekizumab, and 20 (64.5%) received secukinumab.

### 3.2. Patients’ Comorbidities

Patients’ comorbidities are shown in Table 2.

Among the 31 patients, 17 (54.8%) had clinically diagnosed psoriatic arthritis. Overall, 18 patients had hypertension. Ten patients were receiving only antihypertensive treatment, two received only statin therapy, three received antihypertensive and antidiabetic treatment, two received antihypertensive and statin treatment, and three received antihypertensive, antidiabetic, and statin treatment. Enrolled patients had no symptomatic atherosclerosis as a comorbidity before the trial. The median initial body mass index (BMI) was 33.1 (27.2–37.1) kg/m^2^. Nineteen patients (61.3%) from the study population were obese (BMI > 30kg/m^2^) based on the 2013 AHA/ACC/TOS Guideline for the Management of Overweight and Obesity in Adults [28]. Excessive consumption of alcohol was observed in one case; 12 patients (38.7%) were current and seven (22.6%) were former smokers. During the 6-month follow-up period, the patients’ lifestyle and medications that were not related to psoriasis remained unchanged and have been initiated more than 1 year before entering this study. No new medications were allowed.

### 3.3. Clinical Findings

The improvements in patients’ clinical characteristics are shown in Table 3.

The median initial PASI value for the overall study population was 18 (14–24). After 6 months of treatment with anti-IL-17 therapies, the PASI score significantly decreased (*p* < 0.001) to a median value of 0 (0–4). We also found significant changes in the quality-of-life scores (DLQI and EQ VAS). The median initial DLQI and EQ VAS values were 17 (10–28) and 60 (50–80), respectively. In the 6^th^ months of the therapies, DLQI improved significantly (*p* < 0.001) to a median value of 0, and EQ VAS significantly increased (*p* < 0.001) to a median value of 90.

### 3.4. Ultrasonographic Findings

Improvements in the arterial IMTs and the upper limtis of age-adjusted normal values are presented in Table 4 and Table 5. The plaque distributions are presented in Table 6.

Baseline cIMT levels were elevated in 22 of the 31 patients (71%) compared to age-adjusted non-psoriatic healthy controls [29]. Non-calcified plaques in the common carotid arteries were observed in 13 patients, and in the femoral arteries, they were observed in two patients. Calcified plaques were observed in the common carotid arteries in 7 patients. Among these 7 patients, 2 patients also had calcified plaques in their common femoral arteries. After 6 months of therapy, IMT of all three large vessel walls was reduced significantly on both sides; moreover, all the non-calcified plaques improved. The area of the calcified plaques did not change during the observational period. Right cIMT was reduced (*p* < 0.001) from a median value of 1.1 mm to 0.8 mm, and left cIMT was reduced (*p* < 0.001) from a median value of 1.1 mm to 0.7 mm. Right bIMT was reduced (*p* < 0.001) from a median value of 0.75 mm to 0.6 mm, and the left bIMT was reduced (*p* < 0.001) from a median value of 0.8 mm to 0.5 mm. Right fIMT was reduced (*p* < 0.001) from a median value of 0.9 mm to 0.7 mm, and left fIMT was reduced (*p* < 0.001) from a median value from 0.8 mm to 0.7 mm. The cumulative area of the non-calcified plaques in all arteries was reduced, though not significantly (*p* = 0.062), from a median value of 3 mm^2^ to 1.6 mm^2^.

**Figure 1:**
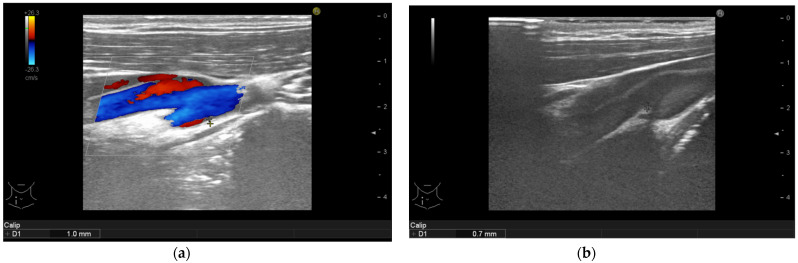
Psoriasis vulgaris. Intima-media thickness measured in right common carotid artery of patient without manifest atherosclerosis before therapy initiation (**a**) and after 6-month of interleukin-17 inhibitor treatment (**b**).

**Figure 2:**
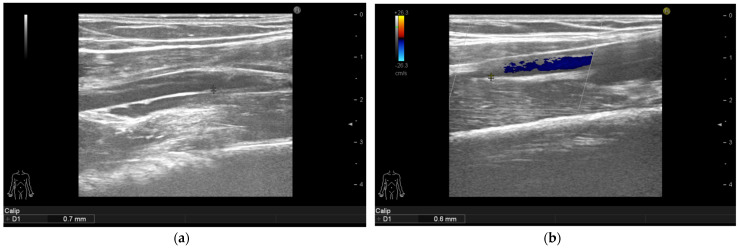
Psoriasis vulgaris. Intima-media thickness measured in right mid-third of the brachial artery of patient without manifest atherosclerosis before therapy initiation (**a**) and after 6 months of interleukin-17 inhibitor treatment (**b**).

**Figure 3:**
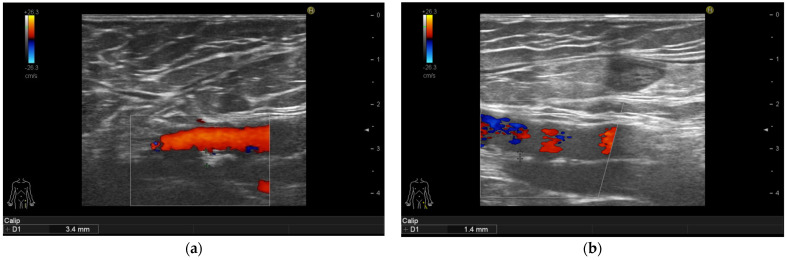
Psoriasis vulgaris. Intima-media thickness measured in left common femoral artery of patient before therapy initiation (**a**) and after 6-month of interleukin-17 inhibitor treatment (**b**).

**Figure 4:**
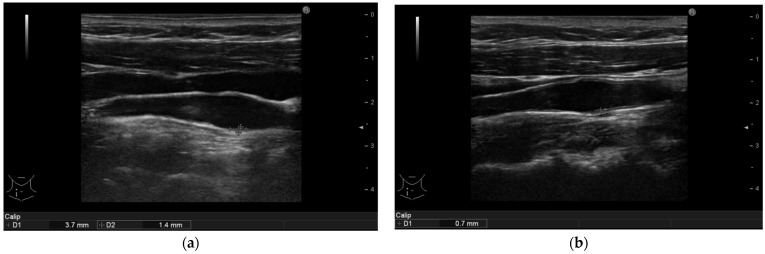
(**a**): Psoriasis vulgaris. Non-calcified plaque measurement in right common carotid artery of patient before therapy initiation. (**b**): After 6 months of interleukin-17 inhibitor treatment the plaque reduced to a no measureable size. The intima-media thickness is indicated.

Ameliorations of the large arteries can be seen in Figure 1, Figure 2, Figure 3 and Figure 4.

Comparing the calcified arteries to the non-calcified ones, we found significant changes both in the baseline and 6-month follow-up parameteres (Table 7). All the baseline and 6-month values were the same or lower in the non-calcified arteries compared to the calcified arteries. The baseline value of the left femoral arteries was significantly lower in the non-calcified arteries. The thickness of the right and left carotid arteries and the left femoral arteries in the 6th month were significantly lower in the non-calcified arteries compared to the calcified arteries. The improvement is more significant in the non-calcified arteries.

### 3.5. Comparing Ultrasonographic Findings between Subgroups of Patients Treated with Secukinumab or Ixekizumab Therapy

Regarding the different pharmacokinetical data and dosage regimens of the two interleukin-17 inhibitors, we compared the two patient groups. There were no significant changes in the two groups regarding the value of the improvement of ultrasonographic findings.

## 4. Discussion

In the ‘psoriatic march’, the inflammatory milieu is responsible not only for skin and joint manifestations, but also for systemic involvement. Thus, psoriasis is accompanied by several comorbidities, such as cardiovascular risk factor metabolic syndrome and its components: diabetes mellitus, hypertension, dyslipidaemia, and obesity [30]. In our study, we enrolled severe, therapy-resistant patients with pronounced skin symptoms; thus, we found examples of all the above mentioned comorbidities. The interpretation of the concomitance of the CVDs and psoriasis is based on the shared immunopathogenic pathway. Th1 cytokines induce atherosclerotic plaque growth, while Th17 cytokines induce intraplaque angiogenesis and hemorrhage [3]. Once the intraarterial surface is injured, blood cells can easily adhere to it, forming a thrombus, which can block blood flow. Severe psoriasis is associated with an estimated excess of 11,500 (95% CI, 1169 to 24 407) MACEs every year in the United States [31]. The prevalence of hypertension among psoriatic patients is higher than that among non-psoriatic healthy controls. The severity of psoriasis influences medication therapy for hypertension control. The parallel use of dual, triple and quadruple antihypertensive drugs is 9.5-fold, 16.5-fold, and 19.9-fold greater, respectively, among psoriatic patients with hypertension than non-psoriatic subjects with hypertension [32,33]. Our study population was similar to those studied by Armstrong et al.: eight patients received dual therapy, two received triple, and one received quintuple antihypertensive therapy. Psoriasis can reduce life expectancy by up to 5 years [7]. Interleukin-17 inhibitor therapy showed a great impact on the flow-mediated dilatation (FMD) in the Evaluation of Cardiovascular Risk Markers in Psoriasis Patients Treated with Secukinumab (CARIMA), according to a study and on the perivascular fat attenuation index (FAI), which are indicators of early atherosclerotic changes [34,35].

In our prospective self-controlled study, 31 adult severe psoriatic patients received secukinumab or ixekizumab therapy. Enrolled patients were the most serious cases: many were receiving second- or the third-line systemic biologic antipsoriatic therapy. Four patients who were bionaive were very serious cases with high PASI and DLQI levels. Previously, all of our patients received systemic traditional therapy, but due to the loss of drug efficacy or primary inefficacy, switching was necessary. The enrolled patients had pronounced skin and/or joint symptoms for at least 3 months, supporting the non-sufficient suppression and existence of systemic inflammation. In this study, we found significant improvements in skin symptoms, quality of life, and carotid-brachial-femoral IMTs. In half of the study population, we found non-calcified plaques, which were also reduced to below-measurable sizes. The treatment has no effect on calcified plaques. Our study supported the hypothesis that moderation of generalized inflammation with anti IL-17 treatment may have a beneficial effect not only on skin and joint manifestations of psoriasis disease but also, through the shared immunopathogenic pathway, on arterial IMTs. This effect is more expressed in the early stage atherosclerotic, non-calcified vessels. Our study group was at increased risk of having CVDs; the patients’ baseline cIMTs were pathologically elevated compared to the age-adjusted non-psoriatic healthy controls, and the majority had non-calcified plaques or manifest atherosclerosis [29].

A possible explanation for our findings is that interleukin-17 inhibitor may have an indirect effect on the vasculature, by reducing the generalized inflammation which is presented also in the arterial wall. Furthermore, results from a murine study show that TGF-β-induced Th17 cells produced IL-17A cytokine, which has an effect on the atheroslcerotic plaque formation, including formation of the collagen cap (expansion of collagen-rich fibrous cap). Based on the authors’ findings, IL-17A has a direct effect on collagen deposition, due to directly promoted procollagen type I expression in cultured smooth muscle cells. IL-17A inhibitor reversed the plaque phenotype associated with IL-17A overproduction in mice with Smad7-deficient T cells [36].

The potential impact of anti-IL-17 agents on cardiovascular morbidity and mortality of severe psoriatic patients needs to be determined in larger studies, as there are limits to using IMT as a surrogate outcome measure. Further investigation is needed to assess the cardiac effect of psoriasis disease and to prepare guidelines for psoriatic patients’ cardiologic screening, as they are at increased risk of MACEs.

## 5. Study Limitations

One limitation of the study is the small sample size. During the follow-up period, we did not stratify patients according to their cardiovascular risk factors. It is difficult to exclude the effects of diabetes mellitus, hypertension, and dyslipidaemia, which also have an impact on arterial IMT levels. The previously used systemic antipsoriatic treatment may also have a delayed effect on the baseline IMT values; however, before the initiation of the applied IL-17 inhibitor therapy, there was an average 3-month “wash out” period. Our patients had several other comorbidities and were taking other medications not related to psoriasis, which is also a limitation of the study; however, patients’ lifestyle and medications remained unchanged throughout the follow-up period, and no new medications were allowed. Non-psoriatic medications were initiated more than 1 year before this study. Progression of atherosclerosis can be determined more accurately with the measurement of endothelial dysfunction in serum samples and/or the assessement of flow mediated dilatation. During our research work, we did not perform measurements regarding functional improvement, but only IMT monitoring.

## Figures and Tables

**Table 1:** Baseline characteristics of severe psoriatic patients treated with interleukin-17 inhibitors.

Patients’ Characteristics	N (%) or Median (IQR)
**Total sample**	31 (100)
**Sex**	
Women	13 (41.9)
Men	18 (58.1)
**Age (years)**	49 (37–62)
**Disease duration (years)**	24 (16–28)
**BMI (kg/m^2^)**	33.1 (27.2–37.1)
**Education**	
Elementary school	3 (9.7)
Secondary school	18 (58.1)
Tertiary Education	10 (32.2)
**Employment**	
Full-time job	17 (54.8)
Retired	4 (12.9)
Unemployed	5 (16.1)
Disability pension	5 (16.1)
**Current smoker**	12 (38.7)
**Former smoker**	7 (22.6)
**Excessive alcohol consumption**	1 (3.2)
**Treatments**	
bionaive	4 (12.9)
Secukinumab	20 (64.5)
Ixekizumab	11 (35.5)

BMI, body mass index; IQR, interquartile range. Bold, separates the different “groups”.

**Table 2:** Comorbidities of severe psoriatic patients treated with interleukin-17 inhibitors.

Comorbidity	N (%)
Psoriatic arthritis	17 (54.8)
Hypertension	18 (58.1)
Diabetes mellitus	6 (19.3)
Bronchial asthma	2 (6.4)
Allergic rhinitis	2 (6.4)
COPD	1 (3.2)
GERD	2 (6.4)
Hyperuricemia	7 (22.6)
BPH	1 (3.2)
Hyperthyroidism	1 (3.2)
Hypothyroidism	2 (6.4)
Psychiatric disorders	4 (12.9)

COPD, chronic obstructive pulmonary disease; GERD, gastroesophageal reflux disease; BPH, benign prostatic hyperplasia.

**Table 3:** Improvement in clinical characteristics of severe psoriatic patients treated with interleukin-17 inhibitors.

Characteristics	Baseline Median (IQR)	6-Month Follow-Up Median (IQR)	*p*-Value
**Clinical Characteristics**			
PASI	18 (14–24)	0 (0–4)	**<0.001**
DLQI	17 (10–28)	0 (0–1)	**<0.001**
EQ VAS	60 (50–80)	90 (85–98)	**<0.001**

PASI, psoriasis area severity index; DLQI, dermatology life quality index; EQ VAS, euroQol visual analogue scale; IQR, interquartile range. Bold, emphase the significant changes.

**Table 4:** Improvement in ultrasonographic examinations of severe psoriatic patients treated with interleukin-17 inhibitors.

Arterial Intima Media Thickness	Baseline Median (IQR)	6-Month Follow-Up Median (IQR)	*p*-Value
Intima media thickness of the right carotid arteries (mm)	1.1 (1.0–1.3)	0.8 (0.6–0.9)	**<0.001**
Intima media thickness of the left carotid arteries (mm)	1.1 (0.9–1.35)	0.7 (0.6–0.9)	**<0.001**
Intima media thickness of the right brachial arteries (mm)	0.75 (0.6–0.9)	0.6 (0.5–0.7)	**<0.001**
Intima media thickness of the left brachial arteries (mm)	0.8 (0.6–0.9)	0.5 (0.5–0.7)	**<0.001**
Intima media thickness of the right femoral arteries (mm)	0.9 (0.8–1.05)	0.7 (0.5–0.9)	**<0.001**
Intima media thickness of the left femoral arteries (mm)	0.8 (0.6–1.1)	0.7 (0.5–0.8)	**<0.001**
Cumulative area of the non-calcified plaques in all arteries (mm^2^)	3.0 (1.8–6.4)	1.6 (1.1–7.5)	0.062

IQR, interquartile range. Bold, emphase the significant changes.

**Table 5:** The upper limits of age-adjusted normal values of non-psoriatic, healthy individuals [29].

Localization	35–39 Years	40–49 Years	50–59 Years	≥60 Years
**Common carotid artery**	0.6 mm	0.64 mm	0.71 mm	0.81 mm
**Bifurcation**	0.83 mm	0.77 mm	0.85 mm	1.05 mm

Bold, separates the different “groups”.

**Table 6:** Plaque distribution in the observed arteries.

	Common Carotid Artery		Brachial Artery		Common Femoral Artery	
	Right	Left	Right	Left	Right	Left
**Number of non-calcified plaques (N)**	7	8	0	0	3	0
**Number of calcified plaques (N)**	4	4	0	0	1	3

Bold, separates the different “groups”.

**Table 7:** Comparing the improvement of calcified and non-calcified arteries of severe psoriatic patients treated with interleukin-17 inhibitors.

	Calcified Arteries		Non-Calcified Arteries		Baseline	6-Month Follow-Up
Arterial Intima Media Thickness	Baseline Median (IQR)	6-Month Follow-Up Median (IQR)	Baseline Median (IQR)	6-Month Follow-Up Median (IQR)	*p*-Value (Mann Whitney U Test) *	*p*-Value (Mann Whitney U Test) *
Intima media thickness of the right carotid arteries (mm)	1.2 (1.0–2.0)	1.0 (0.6–1.1)	1.1 (1.0–1.3)	0.8 (0.6–0.8)	0.385	**0.044**
Intima media thickness of the left carotid arteries (mm)	1.3 (1.3–1.5)	1.0 (0.8–1.1)	1.0 (0.9–1.3)	0.7 (0.6–0.8)	0.054	**0.008**
Intima media thickness of the right brachial arteries (mm)	0.9 (0.7–1.0)	0.6 (0.6–0.8)	0.7 (0.6–0.8)	0.6 (0.5–0.7)	0.200	0.452
Intima media thickness of the left brachial arteries (mm)	0.8 (0.7–0.9)	0.5 (0.5–0.7)	0.8 (0.6–0.9)	0.5 (0.5–0.7)	0.747	0.939
Intima media thickness of the right femoral arteries (mm)	1.4 (0.8–2.2)	0.9 (0.6–1.2)	0.9 (0.8–1.0)	0.7 (0.5–0.9)	0.344	0.136
Intima media thickness of the left femoral arteries (mm)	1.1 (0.9–2.9)	0.8 (0.7–1.1)	0.8 (0.6–1.0)	0.6 (0.5–0.8)	**0.033**	**0.037**
Cumulative area of the non-calcified plaques in all arteries (mm^2^)	-	-	6.0 (n/a)	1.5 (n/a)	-	-

*p ≤ 0.05; n/a = not applicable. Bold, emphase the significant changes.

## Data Availability

The data presented in this study are available on request from the corresponding author. The data are not publicly available in accordance with consent provided by participants on the use of confidential data.

## Abbreviations

CVD	cardiovascular disease
Th cell	T helper cell
IL	interleukin
TNF	tumour necrosis factor
IFN-γ	interferon-gamma
MACE	major cardiac event
MI	myocardial infarct
HR	hazard ratio
CI	confidence interval
IMT	intima media thickness
cIMT	carotid intima media thickness
fIMT	femoral intima media thickness
bIMT	brachial intima media thickness
ICAM-1	intercellular adhesion molecule 1
ApoE	apolipoprotein E
ROR γt	retinoic acid-related orphan receptor γt
PKCß/ERK1/2/NF-κB	protein kinase C beta/extracellular signal-regulated kinase 1/2/nuclear factor kappa-light-chain-enhancer of activated B cells
VCAM-1	vascular cell adhesion molecule 1
QoL	quality of life
PASI	psoriasis area severity index
DLQI	dermatology life quality index
EQ VAS	euroQol visual analogue scale
IQR	interquartile range
MTX	methotrexate
BMI	body mass index
AHA/ACC/TOS	American Heart Association/ American College of Cardiology/ The Obesity Society
FMD	flow mediated dilatation
CARIMA	Evaluation of Cardiovascular Risk Markers in Psoriasis Patients Treated With Secukinumab
FAI	fat attenuation index
TGF-β	transforming growth factor–β
SMAD7	mothers against decapentaplegic homolog 7
